# CoMeT: Configurable Tagged Memory Extension

**DOI:** 10.3390/s21227771

**Published:** 2021-11-22

**Authors:** Jinjae Lee, Derry Pratama, Minjae Kim, Howon Kim, Donghyun Kwon

**Affiliations:** 1 Information Security and AIoT Laboratory, School of Computer Science & Engineering, Pusan National University, Busan 46241, Korea; wlswo3149@gmail.com (J.L.); derryprata@gmail.com (D.P.); rlaalswo201@gmail.com (M.K.); howonkim@gmail.com (H.K.); 2 Computer Security Laboratory, School of Computer Science & Engineering, Pusan National University, Busan 46241, Korea

**Keywords:** memory isolation, tagged memory

## Abstract

Commodity processor architectures are releasing various instruction set extensions to support security solutions for the efficient mitigation of memory vulnerabilities. Among them, tagged memory extension (TME), such as ARM MTE and SPARC ADI, can prevent unauthorized memory access by utilizing tagged memory. However, our analysis found that TME has performance and security issues in practical use. To alleviate these, in this paper, we propose CoMeT, a new instruction set extension for tagged memory. The key idea behind CoMeT is not only to check whether the tag values in the address tag and memory tag are matched, but also to check the access permissions for each tag value. We implemented the prototype of CoMeT on the RISC-V platform. Our evaluation results confirm that CoMeT can be utilized to efficiently implement well-known security solutions, i.e., shadow stack and in-process isolation, without compromising security.

## 1. Introduction

Tagged memory architecture (TMA) is a computer architecture where every memory block has a special memory tag representing the state of the corresponding memory block. TMA has been considered capable of supporting various security solutions [1,2,3,4] depending on how the memory tag is used. For example, developers can use TMA to detect use-after-free vulnerabilities. Specifically, suppose they enforce that the allocated memory objects and freed memory objects have different tag values. In that case, it is possible to identify the memory access to the freed memory object by checking the tag value of the memory to be accessed. Due to this versatility in TMA, commodity processor architectures also announced instruction set extensions for TMA (i.e., memory tagging extension (MTE) [5] in ARM, application data integrity (ADI) [6] in SPARC). For simplicity, we refer to these commercial extensions as tagged memory extensions (TMEs) throughout the rest of this paper. Accordingly, several attempts [7,8] utilize TME to enhance the security of software applications.

Although the detailed implementation is different according to the processor architecture, the common operation of TME is as follows. In TME, the physical and the pointer are augmented to hold the tag. We call the tag for the physical memory a *memory tag* and the tag for the pointer *an address tag*. Specifically, in TME, each physical memory block (e.g., 16-byte memory block in ARM MTE) has its own memory tag. Moreover, memory tags are stored in separate storage (not in the addressable physical memory), so ordinary memory instructions cannot access the memory tag. On the other hand, they locate the tag at the upper end of pointer value in the address tag without introducing additional storage for this. That is, they take advantage of the fact that some of the most significant bits of the pointer are not used for address translation in 64-bit processor architectures. TME only permits the memory operations when the address tag in the pointer and the memory tag corresponding to the target memory address hold the same tag value. Notably, the processor performs the tag comparison operation while processing the memory instructions, so TME does not require additional instructions to check tag values.

However, despite this elaborate design, we found that TME has some limitations when it comes to applying security solutions. First, there is a risk that the security guarantee of TME is incapacitated by an adversary who can tamper with address tags. Memory tags can only be accessed through some special instructions; we can easily stop an adversary from exploiting these instructions by leveraging existing works [9,10]. However, the address tag exists in the upper bits of the pointer value so that the tag value can be altered with ordinary memory and arithmetic instructions through software vulnerabilities (e.g., uninitialized read and integer overflow) [2]. Second, in multi-core systems, since the memory tag exists for each physical memory block, it is impossible to grant different access permissions (i.e., different tag values) for the protected memory region to each core. Lastly, developers cannot configure the detailed access permissions using TME. In MTE, once the address and memory tags hold the same value, they permit read and write access to the target memory region. Thus, developers cannot configure the protected memory region as a read-only memory region using MTE.

Based on these observations, in this paper, we present CoMeT (Configurable Memory Tagging Extension), a new instruction set extension for TMA. At the center of CoMeT, there is a tag permission configuration register (TPCR) to configure the access permissions for each address tag value. Specifically, in CoMeT, the memory access is permitted not only when the address tag and memory tag hold the same value (*tag value check*) but also when the required access permission (readable or writable) for the tag value is set in TPCR (*tag permission check*). Therefore, CoMeT provides the developer with additional functionality to control access to the memory region through TPCR settings even if an attacker modifies the address tag. Additionally, since TPCR is a per-core register that is only accessed through dedicated instructions, developers can safely assign different permissions to each core via TPCR. We built the prototype of CoMeT on RISC-V MTE [11,12] platform. To show the feasibility of CoMeT, we implemented two security solutions, i.e., shadow stack and in-process isolation, using CoMeT. Our evaluation results show that CoMeT can support security solutions more efficiently than TME without compromising security.

## 2. Background

### 2.1. Tagged Memory Extensions

According to our preliminary study, there are two TMEs; ARM Memory Tagging Extension (MTE) [5] and SPARC Application Data Integrity (ADI) [6]. In this subsection, we briefly describe how these extensions.

ARM MTE is an instruction extension newly introduced in ARMv8.5-A architecture. ARM MTE implements a lock and key mechanism [13] to provide a fine-grained access control mechanism for physical memory. In other words, memory access is permitted only when the key value of the pointer variable is the same as the lock value of the target physical memory. Otherwise, an access error is reported. In ARM MTE, the key in the pointer variable is called address tag, and the lock in the physical memory is called memory tag. The memory tag is a 4-bits tag associated with each aligned 16-byte of physical memory. The memory tag is stored in the unaddressable memory, so standard memory instructions cannot access the memory tag. Instead, ARM MTE provides special instructions that can access and update the memory tag, e.g., LDG and STG. The address tag is implemented using the top byte ignore (TBI) feature in ARMv8-A 64-bit architecture. When TBI is enabled, the top byte of the memory address in the pointer is not used for address translation. Thus, in ARM MTE, 4-bits in a top byte of a memory address (i.e., [59:56] bits in 64-bit address) are used for the address tag for the pointer. Similarly, there is a dedicated instruction that assigns a random value to the address tag, i.e., IRG. However, since the address tag resides at the upper bits of the pointer, an attacker can exploit any arithmetic instructions and memory load instructions to manipulate the address tag.

Similar to ARM MTE, SPARC ADI also realizes lock and key mechanism using tagged memory. In SPARK ADI, every 64-bytes of physical memory is associated with a 4-bit memory tag, and the address tag is located at [63:60] bits of the 64-bit address. However, since the address tag is still placed in the top byte of the memory address, there is the same threat to the address tag in SPARC ADI.

### 2.2. RISC-V

RISC-V is an open-source ISA that is widely used in academics and industry at present.

#### 2.2.1. General Registers

RISC-V utilize 32 general registers (x0 to x31). x0 is hardwired with all bits equal to 0, and others are general-purpose registers. Additionally, they are given standardized names, which express their functionality as part of the RISC-V application binary interface (ABI). For example, the x0, x1 and x2 register are called zero, ra and sp, respectively. The x28, x29, x30, x31 register are used for temporary registers, so they are called t3, t4, t5, and t6, respectively.

#### 2.2.2. Control and Status Register

In RISC-V, Control and Status Registers (CSRs) represent various system configurations, such as the base address of the first-level page table (i.e., sptbr). Additionally, RISC-V provides 12-bit encoding space for developers to define and create a custom CSR. Since each privilege level can access the system resources are different, CSRs are also classified according to privilege levels, such as user-level CSRs and supervisor-level CSRs. RISC-V provides instructions for manipulating CSRs; for example, CSRRW and CSRRS/C instructions to read, write, or even set or clear bits on CSR.

#### 2.2.3. RISC-V MTE

**Figure 1:**
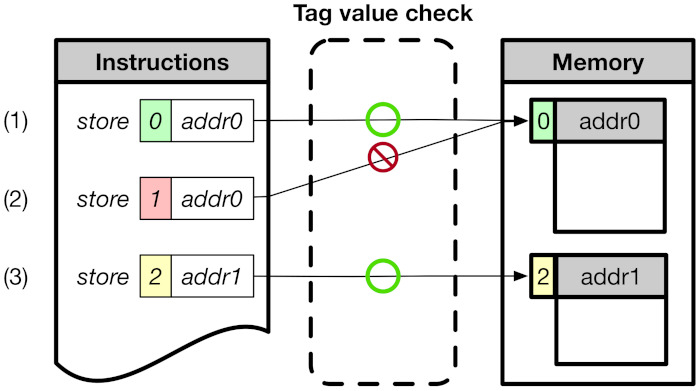
Overview of RISC-V MTE.

As described in Section 1, CoMeT is designed on top of RISC-V memory-tagging extension (RISC-V MTE) [12], which is an open-source project to support tagged memory on the RISC-V processor. RISC-V MTE uses an 8-bit tag for the address tag and memory tag. The address tag resides in the top byte of the 64-bit address, and the memory tag is associated with every 16 bytes of physical memory. In order to manage the memory tag, RISC-V MTE also provides dedicated instructions, i.e., ST (Store Tag) and LT (Load Tag). Figure 1 shows how RISC-V MTE checks the access permission of the tag. (1) and (3) are cases where the address tag value of instructions matches the memory tag value of the corresponding memory area, and access is permitted. (2) is the case where the addresses tag value of instruction mismatch the memory tag value of the corresponding memory area, and access is denied.

## 3. Design

In this section, we explain the design of CoMeT and how CoMeT can be used to realize existing security solutions.

### 3.1. CoMeT

**Figure 2:**
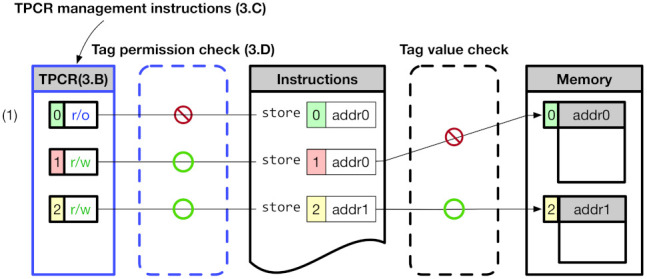
Overview of CoMeT.

Figure 2 shows an overview of CoMeT. The CoMeT provides a tag-based permission control in addition to the RISC-V MTE’s tag-based access control method. The tag value used in the RISC-V MTE has a corresponding permission level to control access permission of an associated memory area in a fine-grained manner. Specifically, in order to change the access permission of the memory, CoMeT utilizes TPCR (Tag Permission Configuration Register) inside each core using dedicated instructions for updating TPCR, without having to access the tag in memory frequently. For example, (1) of Figure 2 shows that store operation to addr0 blocked by tag permission check of CoMeT though address tag value of the store instruction is same with a memory tag value of the memory area pointed by addr0.

### 3.2. Tag Permission Configuration Register

**Figure 3:**
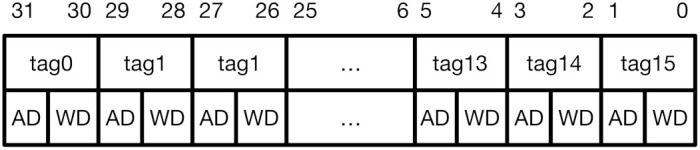
TPCR (Tag Permission Configuration Register).

CoMeT introduces a *tag permission configuration register* (TPCR), a 32-bit user-level CSR, in the system. Figure 3 shows the encoding of TPCR. For each tag, TPCR has 2-bit access permission bits. Each bit in the permission bits indicates Access Disable (AD) and Write Disable (WD), respectively. Thus, when both bits are <0,0>, both read and write operations are possible for the target memory block with the corresponding tag value. If the permission bits are <0,1>, only the read operation is possible. Lastly, when the permission bits are <1,x>, read and write access to the memory block with the corresponding tag value is blocked.

### 3.3. TPCR Management Instructions

**Figure 4:**
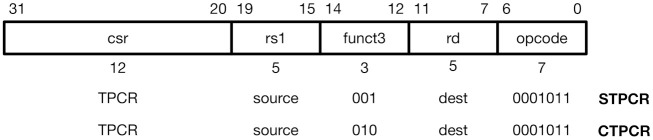
TPCR updating instructions.

CoMeT also provides special instructions to manage TPCR (i.e., STPCR and CTPCR) as shown in Figure 4. STPCR updates the value of TPCR with the value in the source register (rs) and CTPCR clears the value of TPCR by using bitmask in the source register (rs). For example, if the value of the source register (rs) is 0x00000030, STPCR instruction only sets the fifth and sixth bits of TPCR and CTPCR instruction clears these bits. Note that these instructions basically update the value of the destination register (rd) as the zero-extended 64-bit value of TPCR. Thus, a developer can use these instructions to read the original value of TPCR. If the developer does not want to update any destination register, he can configure the destination register as x0, a hardwired-zero register. To avoid unexpected side effects, TPCR cannot be accessed by original CSR manipulating instructions, for example, CSRRW and CSRRS/C instructions. Trying to access TPCR using original CSR manipulating instructions will be detected at the decoding stage of the instruction pipeline and denied.

### 3.4. Tag Permission Check

**Figure 5:**
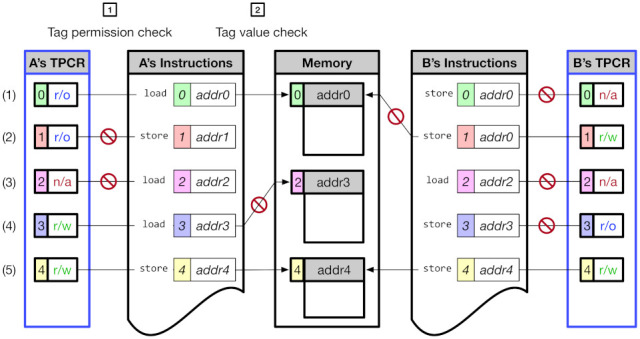
Access Control in CoMeT.

Figure 5 shows several memory operations that can occur in CoMeT. First, CoMeT first checks the tag permission of the address tag. That is, referring to the permission bits of the TPCR, CoMeT checks whether the memory operation is allowed with the corresponding tag value. If the memory operation is performed without required permission, corresponding exceptions occur, and as a result, mcause CSR, which saves the cause of exception in RISC-V, is updated. For example, case (2) raises a ‘store access fault’ (0x0f) exception because the corresponding tag permission is ‘write disable’. Case (3) incurs a ‘load access fault’ (0x0d) due to the ‘access disable’ permission in TPCR. It is notable that a tag permission check can be performed much faster than a tag value check because it can be performed without reading memory tags, as in the case of tag value check. On the other hand, memory access might not be permitted, even if the tag permission check is passed due to the tag value check. For example, in case (4), the memory access is denied because the memory tag value and address tag value do not have the same value. In this case, CoMeT generates synchronous exception ‘instruction page fault’ (0x0c). Consequently, in CoMeT, the memory access is permitted only when both tag permission check and tag value check are passed, as in cases (1) and (5).

Meanwhile, in RISC-V, memory access is also controlled by the permission fields in the page table entry (PTE). Thus, the access permissions for the target memory of TPCR and PTE may not match each other. In this case, CoMeT enforce that the access permission of TPCR does not override the access permission of PTE. For example, if the permission of the TPCR is RW and the permission of the PTE is RO, a write operation to the target memory generates a page permission fault. Note that the advantages of CoMeT compared to this PTE-based access control will be explained in Section 6.

It is notable that each core can assign different tag permissions from those in the other cores because each core has its own TPCR. For example, Figure 5 shows that core A and core B have their own TPCR and assign different access permission on each tag. As a consequence, in CoMeT, developers can enforce different access controls on each core, which is not possible in the existing TME.

### 3.5. Security Solutions

#### 3.5.1. Shadow Stack

The shadow stack [14] is a security solution to protect the return address from software vulnerabilities such as stack buffer overflow. For this, it stores the return address not in the call stack but in another stack (called shadow stack) at the prologue of the function and, in the function epilogue, the return address is retrieved from the shadow stack. Thus, the security of this solution depends on ensuring memory isolation for the shadow stack. Otherwise, an attacker can tamper with the return address in the shadow stack.

**Figure 6:**
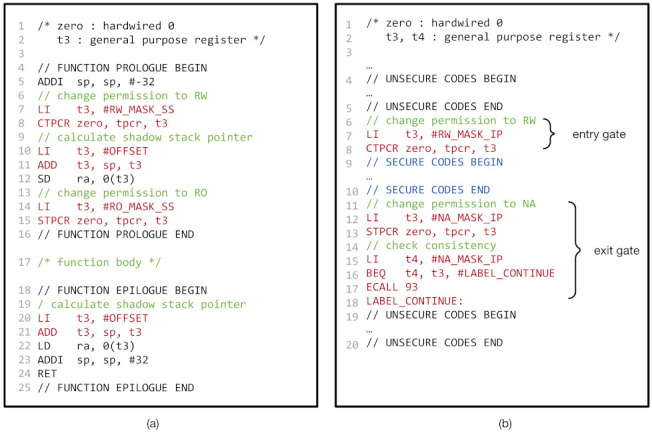
Shadow stack and In-process isolation in CoMeT: (**a**) pseudo code of shadow stack in CoMeT and (**b**) pseudo code of in-process isolation in CoMeT.

Understanding this, we implemented the shadow stack using CoMeT, as follows. First, at loading time, we allocate the memory region for the shadow stack with a dedicated tag value. We locate the shadow stack at a constant offset from the call stack (line 10–11 and 20–21 in Figure 6a). Moreover, we enforce that the tag permission for the tag value is read-only, except for the function prologues. At function prologues, we temporally make the shadow stack writable (line 7–8 and 14–15 in Figure 6a) and store the return address to it. By doing this, we can ensure that any malicious write access to the shadow stack except the function prologues is prevented, since the tag permission for the shadow stack is not writable.

#### 3.5.2. In-Process Isolation

In-process isolation is a well-known security solution that isolates security-critical code and data from other program parts in the same address space. Since it can be applied to various security problems such as kernel extension isolation and browser extension isolation, recently, many security studies [9,10,15] have implemented it with several hardware features.

Thus, we also enforced the in-process isolation with CoMeT as follows. Firstly, we placed the security-sensitive data in the memory region tagging with a dedicated value (e.g., TAG1 in Figure 6b). Then, we configured TPCR so that tag permission for the dedicated value was readable and writable only when the security-critical code is executed (line 7–8 and line 12–18 in Figure 6b). In other words, the tag permission of the value should not be accessible when the other code is executed.

## 4. Evaluation

In this section, we evaluated two aspects of CoMeT. First, we verified that CoMeT can block the memory access with inadequate tag permission. Then, we measured the performance results when security solutions are implemented with CoMeT and existing TME. To apply security solution to the software, we used LLVM Clang-12 project [16] and GCC toolchains (riscv64-unknown-linux-gnu and riscv64-unknown-elf).

### 4.1. Functionality Verification

For the functionality verification, we implemented the prototype of CoMeT on top of RISC-V MTE implementation [12] using QEMU 4.11. The RISC-V MTE supports key features of TME, such as top-byte ignore, memory region for storing tags, and custom instructions for loading and storing tags. However, TME uses 4-bit tags, so we changed RISC-V MTE to use 4-bit tags (initially, it used 8-bit tags). Furthermore, to support CoMeT, we added TPCR to facilitate the tag permission check, and different exceptions for each access violation on QEMU, and modified LLVM to support TPCR manipulating instructions.

We verified the tag permission check of CoMeT by inserting memory access instructions that do not have adequate tag permission to the memory region. For example, we inserted store instructions, which stores source register data to the memory region assigned read-only access permission by RO_MASK_SS to lines between line 16 and line 17 of Figure 6a. Consequently, since access permission to the memory region is configured as read-only at line 15, we confirmed that CoMeT could detect such access by generating a ‘store access fault’ exception.

### 4.2. Performance Evaluation

Since QEMU is not cycle-accurate, to evaluate the performance impact of CoMeT, we used a PolarFire SoC Icicle Kit [11] that supports a 64-bit RISC-V processor with RV64IMAC extensions. Linux 5.6.16 was used as a host operating system. Unfortunately, however, we were unable to apply TME and CoMeT on this board as we could not modify the board’s hardware. Thus, we conducted a performance experiment using the following proxy measurement method.

#### 4.2.1. Proxy Measurement

Each time a memory access instruction is executed, TME reads the memory tag of the corresponding memory region to perform a tag value check for the address tag. To emulate this tag load operation, we inserted a memory load instruction before all memory access instructions. Undoubtedly, this emulation will be insufficient to measure the actual performance overhead caused by real tagged memory. However, since the performance overhead caused by tagged memory is common to CoMeT and TME, even if the overhead is underestimated, the relative performance differences between CoMeT and TME would still be valid. On the other hand, to emulate the performance overhead due to added instructions to access TPCR, we replaced these instructions with existing CSR access instructions. Since TPCR is also one of the CSRs, we believe that this emulation shows almost the same performance as the actual TPCR access instruction. We implemented a function to trigger and stop the measurement. The CPU cycle measurement is carried out by reading the CPU cycle count CSR on RISC-V called cycle on each start_trigger() function. Then on stop_trigger() we reread the CPU cycle count and subtracted it using the CPU cycle count at start_trigger().

#### 4.2.2. Shadow Stack Using TME

**Figure 7:**
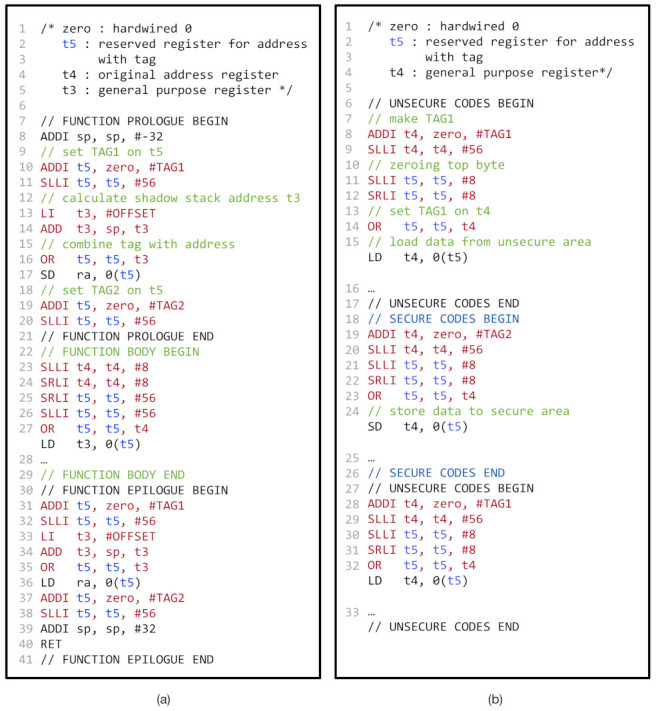
Shadow stack and In-process isolation in TME: (**a**) pseudo code of shadow stack in TME and (**b**) pseudo code of in-process isolation in TME.

For the performance comparison, we implemented the shadow stack with TME, as shown in Figure 7a. Similar to the shadow stack with CoMeT (as described in Section 3.5.1), we allocated the memory region for the shadow stack with a dedicated tag value (e.g., TAG1 in Figure 7a). However, for the same security guarantee, we introduced additional code instrumentation to ensure that the address tag is not manipulated by attackers. Unlike CoMeT, TME has no additional access control mechanism to prevent memory access to the shadow stack region with manipulated address tag. Thus, we reserved a general-purpose register (e.g., t5 in Figure 7a) for the memory address register in all memory access instructions in the program. As a consequence, since an attacker cannot tamper the reserved register with software vulnerabilities, we can prevent malicious access to the address tag in the reserved register. To reserve the register, we changed the LLVM compiler to not use the reserved register for other instructions and recompiled the C library using –with-target-cflags=-ffixed-reg option.

#### 4.2.3. In-Process Isolation Using TME

For the performance comparison, we also implemented the in-process isolation with TME, as shown in Figure 7b. We initially place the security-sensitive data and other data in the memory regions tagging with different tag values (e.g., TAG2 and TAG1 in Figure 7b). Then, like Figure 7a, we reserved a general-purpose register (e.g., t5 in Figure 7b) for the memory address register in all memory access instructions. Lastly, we instrumented the program so that the address tag of the reserved register held the proper tag value. For example, in Figure 7b, line 8–14 and 28–32 show the instructions to enforce the address tag of t5 register holds TAG1 value, and line 19–23 show the instructions that configure the address tag as TAG2.

### 4.3. Experimental Result

**Figure 8:**
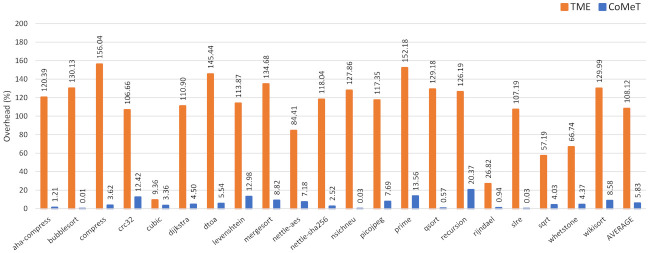
Execution time overhead for the shadow stack with CoMeT and TME.

Figure 8 shows the performance overhead when the shadow stacks using CoMeT and TME are applied to benchmarks in BEEBs benchmark suite [17]. As a result, compared to the baseline, the average overheads of CoMeT and TME are 5.83% and 108.12%, respectively. The main reason for this result is that the number of memory instructions required for instrumentation in the two shadow stack implementations is different. To be specific, in TME, all memory access instructions have to be instrumented to hold a dedicated address tag value. However, in CoMeT, just memory access instructions to the shadow stack in the function prologues need to be instrumented, thanks to TPCR.

**Figure 9:**
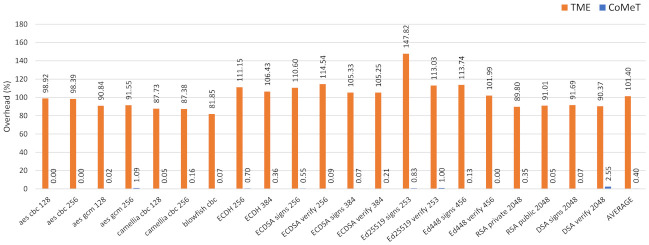
Execution time overhead for the in-process isolation with CoMeT and TME.

We also conducted a performance evaluation for the in-process isolation. Specifically, we applied in-process isolation mechanisms using CoMeT and TME to cryptographic functions in OpenSSL crypto library 1.1.1l. As shown in Figure 9, compared to the baseline, the average overhead of CoMeT and TME are 0.4% and 101.4%, respectively. Similar to the result of the shadow stack, this is because TME needs to manipulate the tag always before accessing memory. On the other hand, in CoMeT, only the tag permission is changed at the beginning and end of the security-critical code with the help of TPCR. Consequently, with the same level of security, TME incurs much more performance overhead than CoMeT.

## 5. Related Work

### 5.1. Tagged Architecture

Numerous works [1,2,3,4] using tagged memory have already been proposed CoMeT. Loki [1] is similar to CoMeT in that it checks access permissions according to tag values. However, since Loki does not use the address tag, they define the legitimate code that can access the specific memory tag value as a program or module, not an instruction. In addition, Loki associates a 32-bit memory tag for each 32-bit physical memory word, resulting in higher memory cost than CoMeT. Meanwhile, HDFI [2] proposed a tagged architecture with low memory overhead by using 1-bit memory tags per memory word. However, HDFI only verifies the memory tag when loading memory, so HDFI is inadequate when the security solution needs to check the memory writes. CHERI [3] proposes a capability model that defines the bounds and access permission of the memory region accessed by each pointer instead of using the address tag. To protect the integrity of capabilities in the memory, CHERI uses tagged memory. Compared to CoMeT, CHERI requires a significant change in the processor architecture (e.g., register files and instructions for the capability management). Similar to CoMeT, TIMBER-V [4] proposed a tagged memory architecture on the RISC-V platform. However, they used he tagged memory to isolate four security domains.

### 5.2. Instruction Extensions for Memory Protection

CoMeT is an instruction extension for memory protection using tagged memory. Of course, before CoMeT, there were various instruction extensions for memory protection. First, ARM MTE [5] and SPARC ADI [6] are extensions for the tagged memory like CoMeT. However, as described in Section 1, these extensions have some limitations when applied to security solutions, and CoMeT is designed to overcome them. Second, some recent studies [9,10] have proposed in-process isolation techniques using Intel memory protection keys (MPK). In MPK, each memory page is associated with a 4-bit key value, and the user process can control the access permission for the memory pages that hold the same key value by configuring the protection key rights register (PKRU). Similar to MPK, CoMeT also introduces a special register called TPCR that can be accessed by a user process. However, CoMeT’s TPCR is different from PKRU in that it sets access permissions to memory blocks having the same memory tag. Notably, in CoMeT, the block size is a 16-byte, which is much smaller than the size of the memory page. This enables CoMeT to support various security solutions, such as temporal memory safety, that require a protection mechanism for each memory object, which is difficult to implement with MPK. Lastly, similar to Intel MPK, ARM domain [5] add a domain ID to the first-level page table entry and controls access rights to memory regions with the same domain ID through the domain access control register (DACR). Thus, security studies for fault isolation [18,19] and in-process isolation [15] have utilized the ARM memory domain. However, DACR is only accessible in the OS kernel, and the ARM memory domain is no longer available in 64-bit ARM architecture.

## 6. Discussion

### 6.1. The Threat against Address Tag

As explained in Section 1, in TME, since the address tag is located in the upper bits of the pointer, there is a risk of being tampered with general arithmetic instructions or memory instructions. CoMeT is also designed in addition to RISC-V MTE, so CoMeT is also exposed to these threats. In order to protect the address tag from such threats, a possible way would be to manage the address tag in separate storage, as in the case of the memory tag. However, this will introduce significant changes in the processor architecture. Instead, in CoMeT, we add an additional access control process called tag permission check so that, even if an attacker modifies the address tag, access to the protected memory region can be prohibited.

### 6.2. Comparison with Page Table Based Access Control

Typically, modern processor architectures already provide access control mechanisms using permission bits in the page table entry. Compared to this mechanism, CoMeT has the following two advantages. First, CoMeT can configure the protected memory regions in a more fine-grained way. Specifically, in the prototype of CoMeT, a memory tag is associated with a 16-byte physical memory block, so that developers can grant different access permissions to each 16-byte block. On the other hand, the page table can configure the permission bits in the page granularity, i.e., 4 KB. Additionally developers can configure the memory permissions of CoMeT without the intervention of the OS kernel because memory tags and TPCR are accessible in the user process. On the other hand, the page table is managed by the OS kernel; therefore, to change the permission bits, a system-level function, e.g., mprotect, should be invoked. These functions include a system call, which typically consumes hundreds of CPU cycles.

## 7. Conclusions

In this paper, we presented CoMeT, a new tagged memory extension on RISC-V. In CoMeT, whenever the memory operation performs, the processor checks the access permission of the value in the address tag before comparing values in the address tag and the memory tag. For this, CoMeT introduces TPCR, a special CSR for representing access permissions for each tag value, and dedicated instructions for managing TPCR into the instruction set architecture. Thanks to this tag permission check mechanism, CoMeT can efficiently prevent malicious access to the sensitive memory region even if an attacker manipulates the address tag residing in the upper bits of the pointer to bypass the tag comparison mechanism of TME. The evaluation results show that CoMeT can support well-known security solutions in a highly efficient manner.

## Data Availability

Data for the experiments are available from the authors on request.

## References

[1] Zeldovich N., Kannan H., Dalton M., Kozyrakis C. Hardware Enforcement of Application Security Policies Using Tagged Memory. Proceedings of the 8th USENIX Symposium on Operating Systems Design and Implementation (OSDI 08).

[2] Song C., Moon H., Alam M., Yun I., Lee B., Kim T., Lee W., Paek Y. HDFI: Hardware-assisted data-flow isolation. Proceedings of the 2016 IEEE Symposium on Security and Privacy (SP).

[3] Woodruff J., Watson R.N., Chisnall D., Moore S.W., Anderson J., Davis B., Laurie B., Neumann P.G., Norton R., Roe M. The CHERI capability model: Revisiting RISC in an age of risk. Proceedings of the 2014 ACM/IEEE 41st International Symposium on Computer Architecture (ISCA).

[4] Weiser S., Werner M., Brasser F., Malenko M., Mangard S., Sadeghi A.R. TIMBER-V: Tag-Isolated Memory Bringing Fine-grained Enclaves to RISC-V. Proceedings of the Network and Distributed System Security (NDSS) Symposium 2019.

[5] Seal D. (2001). ARM Architecture Reference Manual.

[6] Aingaran K., Jairath S., Konstadinidis G., Leung S., Loewenstein P., McAllister C., Phillips S., Radovic Z., Sivaramakrishnan R., Smentek D. (2015). M7: Oracle’s Next-Generation Sparc Processor. IEEE Micro.

[7] Serebryany K. (2019). ARM Memory Tagging Extension and How It Improves C/C++ Memory Safety. Login USENIX Mag..

[8] Tagged Pointers in Android. https://source.android.com/devices/tech/debug/tagged-pointers.

[9] Park S., Lee S., Xu W., Moon H., Kim T. libmpk: Software abstraction for intel memory protection keys (intel MPK). Proceedings of the 2019 USENIX Annual Technical Conference (USENIX ATC 19).

[10] Vahldiek-Oberwagner A., Elnikety E., Duarte N.O., Sammler M., Druschel P., Garg D. ERIM: Secure, efficient in-process isolation with protection keys (MPK). Proceedings of the 28th USENIX Security Symposium (USENIX Security 19).

[11] PolarFire SoC FPGA. https://www.microsemi.com/existing-parts/parts/152514.

[12] Gattaca-Lab (2020). RISC-V MTE. https://github.com/gattaca-lab/riscv_mte.

[13] Frascino V. ARM v8. 5 Memory Tagging Extension. Proceedings of the Linux Plumbers Conference.

[14] Burow N., Zhang X., Payer M. SoK: Shining light on shadow stacks. Proceedings of the 2019 IEEE Symposium on Security and Privacy (SP).

[15] Chen Y., Reymondjohnson S., Sun Z., Lu L. Shreds: Fine-grained execution units with private memory. Proceedings of the 2016 IEEE Symposium on Security and Privacy (SP).

[16] Lattner C. LLVM and Clang: Next generation compiler technology. Proceedings of the BSD Conference.

[17] Pallister J., Hollis S.J., Bennett J. (2013). BEEBS: Open Benchmarks for Energy Measurements on Embedded Platforms. arXiv.

[18] Zhou Y., Wang X., Chen Y., Wang Z. Armlock: Hardware-based fault isolation for arm. Proceedings of the 2014 ACM SIGSAC Conference on Computer and Communications Security.

[19] Manès V.J., Jang D., Ryu C., Kang B.B. (2018). Domain Isolated Kernel: A lightweight sandbox for untrusted kernel extensions. Comput. Secur..

